# NMR Metabolomics Applied on the Discrimination of Variables Influencing Tomato (*Solanum lycopersicum*)

**DOI:** 10.3390/molecules25163738

**Published:** 2020-08-16

**Authors:** Ana Cristina Abreu, Ignacio Fernández

**Affiliations:** Department of Chemistry and Physics, Research Centre CIAIMBITAL, University of Almería, Ctra. Sacramento, 04120 Almería, Spain; acabreu@ual.es

**Keywords:** NMR metabolomics, multivariate data analysis, chemometrics, tomato, Solanum lycopersicum

## Abstract

Tomato composition and nutritional value are attracting increasing attention and interest from both consumers and producers. The interest in enhancing fruits’ quality with respect to beneficious nutrients and flavor/aroma components is based not only in their economic added value but also in their implications involving organoleptic and healthy properties and has generated considerable research interest among nutraceutical and horticultural industries. The present article reviews up to March 2020 some of the most relevant studies based on the application of NMR coupled to multivariate statistical analysis that have addressed the investigation on tomato (*Solanum lycopersicum*). Specifically, the NMR untargeted technique in the agri-food sector can generate comprehensive data on metabolic networks and is paving the way towards the understanding of variables affecting tomato crops and composition such as origin, variety, salt-water irrigation, cultivation techniques, stage of development, among many others. Such knowledge is helpful to improve fruit quality through cultural practices that divert the metabolism towards the desired pathways and, probably more importantly, drives further efforts towards the differentiation of those crops developed under controlled and desired agronomical conditions.

## 1. Introduction

Plant systems are remarkably complex and show an outstanding inherent diversity as a result of complex networks of biochemical pathways, which represent the phenotypic outcome of the genomic makeup, transcript expression and protein function (enzyme activity, kinetics, substrate/cofactor availability, etc.) [1]. Understanding the biochemistry and regulation of specific metabolic pathways have been the aim of hundreds of studies over several centuries, but only in the last two decades, with the availability of the Omics platforms and the development and optimization of new technologies, it was possible to capture the organism’s metabolic changes in a multipicture form and in a global and untargeted manner [2]. In the Omics world, metabolomics, also termed metabolic profiling, is the non-targeted study of global changes in small-molecule metabolites, and aims to improve the understanding of the complex metabolic networks and the subsequent physiology and biochemical composition of the system under study [3]. The integration of metabolic profiling with other omics tools has proven to be highly effective for functional gene identification and pathway elucidation in plant primary and secondary metabolism [4].

NMR spectroscopy is a unique, powerful, and reliable tool in natural product structure determination in metabolomics studies. It combines a high-throughput analysis of a large number of metabolites and high structural elucidation capabilities with its intrinsic quantitative nature and straightforward sample preparation [5,6]. Besides, NMR spectroscopy yields highly reproducible data, does not require separation of compounds from biological mixtures and is noninvasive. NMR sensitivity may have restricted its use as a method of choice in the past, in particular when compared to another widely used technique such as mass spectrometry (MS), but it has progressed with recent technological developments, including improvements in hardware (e.g., high-resolution NMR, the use of cryoprobes, micro and in-flow probes), in pulse sequences and spectral acquisition and post-processing protocols. Thus, it is not surprising that NMR has now become the predominant untargeted metabolomic profiling method in a wide range of scientific domains in plant sciences, including pharmacology, chemotaxonomy, plant functional genomics, plant stress physiology, plant pathology, and green biotechnology [7]. On the more technological or innovation side of the whole chain of research and development, metabolomics has also attracted much attention particularly in the field of food science, to assess food quality, safety, processing, and postharvest [8,9]. NMR metabolomics has been applied to profile the impact of time, stress, nutritional status, and environmental perturbations on complex mixtures of plant metabolites and result in vast and complex data sets [3,10]. Comprehensive analysis of such data sets using adapted statistical methods and modeling has opened up the possibility of obtaining single or combinations of metabolites as markers, which may serve for diagnosis or prediction of specific key traits, disease outbreak frequency, developmental stages, food sensory evaluation, crop yield, etc. [11].

**Figure 1:**
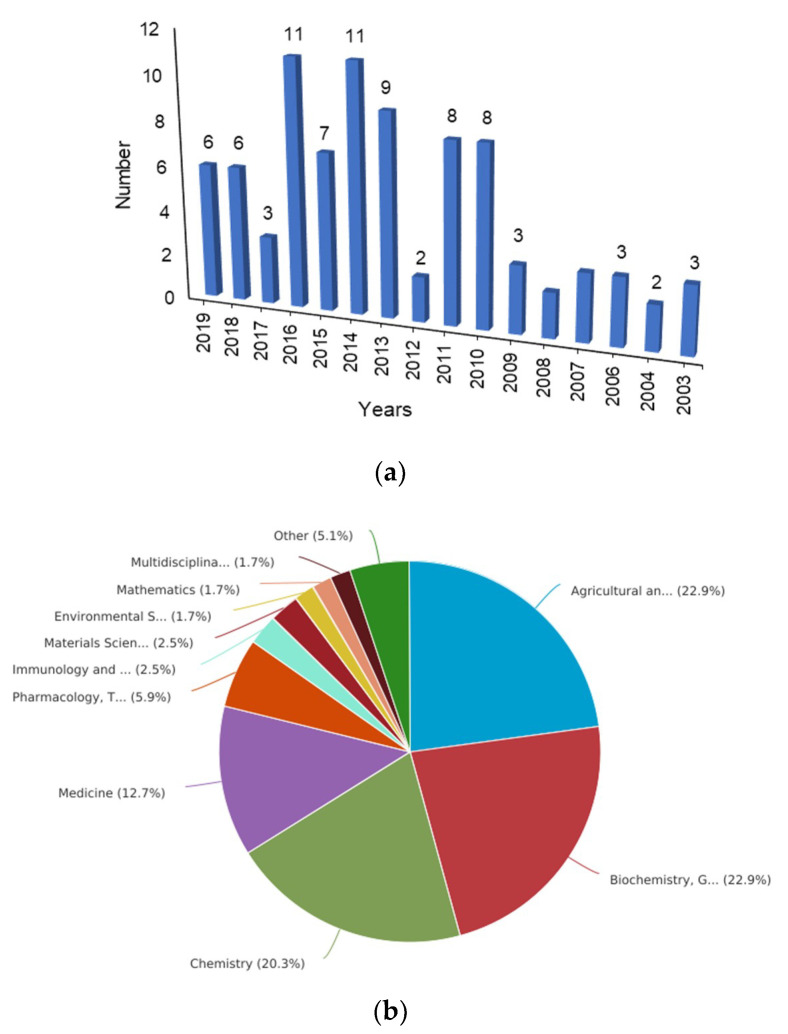
(**a**) Evolution of publications (articles, reviews and book chapters) on NMR metabolomics in tomato research between 2003 and 2019. The number of contributions as a function of year was obtained by using Web of Science and using the words “NMR metabolomics” and “tomato” as topic; (**b**) Scopus search results per subject area using the words “NMR metabolomics” and “tomato” both as Article title, abstract, or keyword.

Production of fruit, which is essential in human nutrition, is recently under significant pressure not only from environmental stresses but also from changes in consumer preference for taste and nutritional value [12]. Extensive metabolomics analyses have been carried out to understand the mechanisms linking metabolism to fruit phenotypes, which will help to improve breeding strategies and crop traits, such as fruit quality, nutritional value and flavor, and to evaluate the influence of fruit storage, transport, and processing on these traits [13]. Tomato (*Solanum lycopersicum*) has quickly become a favored species for metabolomics research. It fills a niche that cannot be occupied by *Arabidopsis* regarding studies on fleshy fruits [13]. Tomato is both an economically important crop, as one of the world’s most important and widely grown and consumed vegetables [14], and it is a model plant species, due to its diploid, relatively compact, recently sequenced genome and its large genetic and genomic resources [15]. These factors are a significant driving force behind this fruit research, which has shown vast recent progress towards understanding the gene regulatory circuitry and metabolic changes [16]. The increasing interest towards application of NMR in food science is revealed both by the constantly increasing number of papers and reviews on this topic and by international and national congresses dedicated to this field [17]. Figure 1a shows the increasing number of NMR metabolomic studies on tomato in the last two decades. Many of these studies have specifically been focused on traits relevant to the food and agro-industries. In this regard, Figure 1b shows the distribution of these articles as a function of subject areas.

In this review, we highlight the progresses made in tomato metabolomics where ^1^H-NMR has been used as the metabolic profiling technique. Some important aspects concerning the design and methodology of an NMR metabolomics study in plant and food science are given and focusing on tomato fruit characterization. An overview of the current literature data was made, evidencing the usefulness of NMR metabolite profiling in the investigation of tomato variety discrimination, fruit development, ripening, quality, geographical origin, and daily and seasonal changes. Changes in genotype and phenotype of tomato fruit have also been widely exploited with metabolomic approaches to obtain information regarding fundamental aspects of plant physiology, fruit growth, and development [13] and are reviewed herein. Recent applications of NMR metabolomics involving metabolic markers as tools for monitoring or predicting plant performance and plant responses to agronomic and environmental conditions and to abiotic and biotic stresses are highlighted. Finally, its use in the food industry for quality control of tomato-derived products, such as tomato sauces, are emerging and are briefly discussed. In addition to proton ^1^H NMR (in both liquid- and solid-sates), the usefulness of NMR relaxometry and imaging for studying tomato development and ripening processes is also evidenced in this review. All of the studies here described have already considerably expanded our knowledge and continue to do so concerning many aspects of tomato plant and fruit, both visible and chemical.

## 2. Metabolomics Platform in Plant Sciences

### 2.1. NMR Suitability in Plant and Fruit Metabolomics

Metabolomics are like a snapshot of an organism, allowing a non-biased identification and quantification of the metabolites present in a given matrix at a given time point. Metabolic profiling, in terms of primary and secondary metabolism, is increasingly becoming popular in assessing plant phenotypes, fruit development, genetic diversity, and as a diagnostics tool of a plant status, with direct relationship to the exhibited visual characteristics (phenotype). All changes related to long-term or induced processes, may be readily detected by a metabolomic approach. On the contrary, to observe fast metabolic changes such as the immediate secondary metabolite production in a plant after wounding, it is necessary to measure the fluxes (i.e., fluxomics) rather than the metabolites concentrations, which can be done with magnetically active isotopes but isotopically enriched such as ^13^C.

Generally, a plant metabolomics study can be extremely complex due to the immense diversity of metabolite chemical structures present in plants, especially among the secondary metabolites which are specific for each species, and that largely vary depending on the plant tissue, developmental stage, and environmental conditions. Recently, using a few robust and rapid analytical systems, highly valuable biological information can be discerned from steady-state semiquantitative measurements of a tractable number of compounds (i.e., a few hundred) and samples (e.g., a few dozen). Metabolite screening requires maximum sensitivity with a broad compound coverage. Also, in order to avoid biases against certain classes of compounds, untargeted analytical methodologies are required. The expansion of metabolomic technologies resulted in the usage of a diverse range and configuration of instruments and analytical methods. Mostly MS technologies are used, but also other techniques such as liquid chromatography (LC) coupled to photo diode array (PDA), infrared and Raman spectroscopy have been applied in plant metabolomics. Among the untargeted analytical methodologies used to analyze fruits, NMR has recently gained an important role because it can simultaneously bring “high-throughput” spectroscopic/structural information on a wide range of metabolites with a high analytical precision [18]. For instance, tomato peel extracts contain, besides all-*E* lycopene as the major carotenoid, various *Z*-stereoisomers at a content of ca. 13% of the total carotenoid content, where the hyphenation of HPLC with NMR as detector allowed their identification 9-*Z*, 13-*Z*, 9,13-*ZZ,* and 9,13′-*ZZ* geometrical isomers. Unfortunately, *Z*/*E* stereoisomers lead to the same mass spectra and fragmentation patters, even with LC-MS, and therefore they are not distinguishable by using HPLC-MS [19,20].

In NMR metabolomics, usually the most sensitive and commonly occurring magnetic nucleus (i.e., ^1^H) is observed. However, more information on topics such as metabolite flux can be obtained with other nuclei, particularly ^13^C and ^15^N [21]. Routine NMR “omic” analytical methods suffer from several drawbacks, not so much its low sensitivity anymore, which has shown to considerably increase in the last decade, but mostly due to high signal overlap in complex spectra. The ^1^H-NMR spectra of fruit and plant extracts are inevitably crowded and hampers the identification and quantification of metabolites. Nevertheless, there are strategies that allow to overcome signal overlap, which can be obtained by spreading the resonances in a second dimension using 2D NMR spectroscopy, or by applying specific filters such as CPMG or diffusion modules. The former removes signals with short transverse (T2) times, i.e., proteic or polypeptidic entities, whereas the latter eliminates signals coming from very low molecular weight species such as solvent molecules, with the additional advantage that does not perturb the overlapped signals. Successful applications of quantitative 2D NMR in the field of metabolomics have been reported [22,23,24]. In particular, heteronuclear single-quantum coherence (HSQC) experiments can be used to determine whether or not the usual suspects (primary metabolites and common secondary metabolites in plants) are present and it also allows metabolite quantification [25]. Still, the use of quantitative 2D NMR in metabolomic studies is limited by the longer acquisition time.

### 2.2. Design an NMR-Based Metabolomics Study in Plant Science

**Figure 2:**
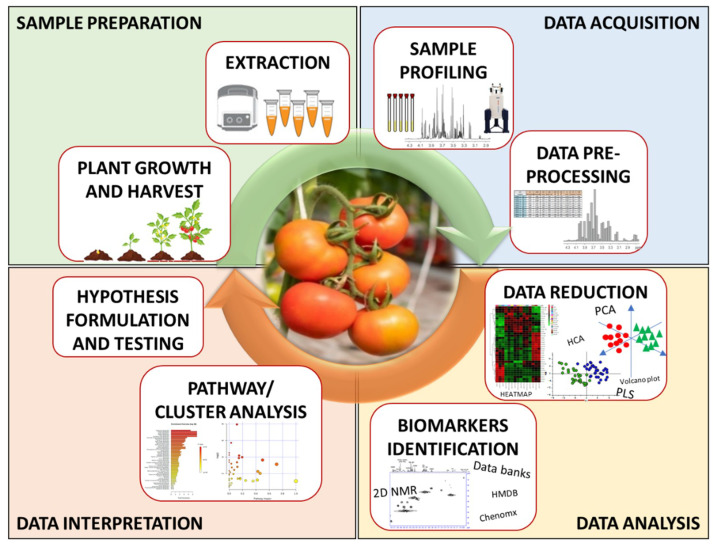
A general workflow of an NMR-based metabolomics study in plant sciences.

A metabolomics experiment will fail at the first hurdle if not properly thought out and designed. While results will almost always be obtained, their reliability and biological interpretation may be motive of doubt if the approach has not been correct [26]. The experimental design should ensure that the analytical data derived from the collected biological material would allow answering the initially proposed biological question through a reliable statistical analysis [27]. After a careful experiment design, the ^1^H-NMR metabolomic process can be envisioned as four steps, including: (1) sample and extract preparation, (2) spectra acquisition, (3) spectra and data processing and analysis, and, finally, (4) data interpretation. Figure 2 shows the workflow of tasks that constitute a plant-related NMR metabolomics study.

The first step is therefore a good experiment design. Important aspects such as sample size, sample pooling and number of biological and technical replicates that will ultimately determine the robustness of the dataset and its suitability for subsequent statistical (multivariate) data analysis, should be considered. The number of samples and/or size of the groups needed for a metabolomics experiment depends on the biological variability associated with the system being studied compared with the analytical variability of the analytical platform [28]. According to the number of samples, three categories of sample series can be roughly defined: small (fewer than 50 samples), average (50 to 200 samples) and large size (over 200 samples) [25]. The larger the number of samples, the easier may be the identification of patterns or metabolite markers that are characteristic for a species, a cultivar, stage of development, or conditions, such as disease state or stress [25]. In this section, we will briefly discuss some important aspects to take in consideration when developing a plant-related metabolomics study.

#### 2.2.1. Sample Provision and Preparation

Sample provision and preparation, their handling and storage, as well as the actual performance of the extraction and analysis itself, are also of critical importance and greatly affect the reliability and the significance of the biological interpretation of the metabolomics results [26]. Minor changes in sample collection, extraction, or storage greatly affect metabolite stability and hence can lead to major changes in the observed metabolome. The ultimate aim is thus to minimize these biologically irrelevant changes. Metabolomics samples have to be collected uniformly using rapid procedures, i.e., freezing foliar samples in liquid nitrogen, in order to avoid changes due to fast enzymatic turnover rate. Improper handling of biological samples is the most likely source of bias in metabolomic studies [29]. Some important aspects concerning sample collection were vastly described by several authors, such as Rodrigues et al. [27] and Barnes et al. [28]. For instance, Barnes et al. [28] refers that excessive variation between groups caused by, for example, a lack of control on the effect of diet or the time of day of sample collection can masquerade biologically relevant changes in metabolite levels and affect the reproducibility of the data. If working under growth chambers and other controlled environmental conditions, plants should be rotated during the experiment to avoid interferences in their metabolism caused by variations in light intensity or ventilation [27]. If plants are grown in a greenhouse or field conditions, variation in environmental conditions are also expected to occur, so an usual strategy is to arrange plants in a block design, and assign randomly the treatments within each block [27,30]. Then, comparison between treatments are performed within blocks, since the variability within each block is lower than the variability between blocks. Randomization is thus critical for reducing experimental error and biological variability [27].

Several extensive reviews addressing sample preparation and data acquisition in more detail can be found elsewhere [27,31,32,33]. A minimal and simple sample preparation protocol has various advantages in terms of speed, capability, consistency, reproducibility, robustness, and efficiency. As water is the main component of plant tissue, and its two protons nuclei are NMR detected, it is important to remove it by freeze-drying. To be more efficient, this step should be performed after cryogrinding, since fine plant powder facilitates the freeze-drying process and also the extraction. Samples should be stored preferentially at −80 °C and ideally for less than 6 months. Nevertheless, their stability during storage needs to be determined [8]. Sample preparation and data acquisition should be done over a short period of time (1 to 2 weeks), and preferably continuously (less than 1-month gap) [8].

**Figure 3:**
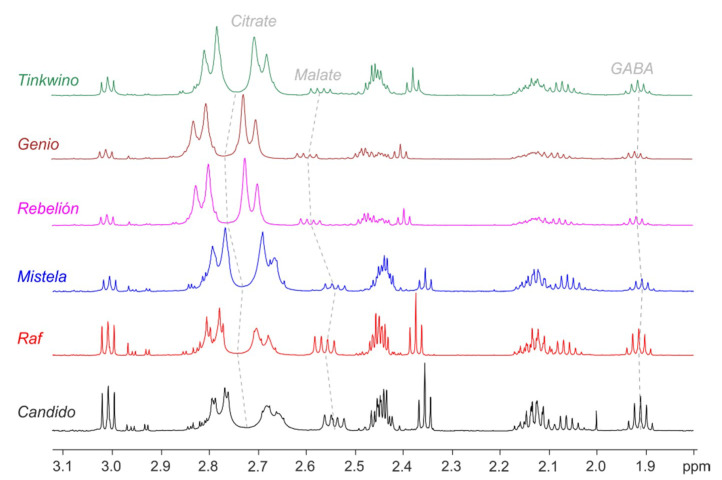
^1^H NMR spectra obtained from CD_3_OD: D_2_O KH_2_PO_4_ buffer at pH 6.0 (50:50, *v*/*v*) extracts of tomatoes from Almeria province (Spain) showing varying differences in chemical shifts and splitting resolution, i.e., citrate, malate, or γ-aminobutyric acid (GABA). Spectra were recorded using a presaturation pulse sequence (Bruker 1D noesygppr1d) in a Bruker Avance III 600 spectrometer operating at a proton frequency of 600.13 MHz at 293 ± 0.1 K, using a 5 mm quadruple resonance inverse cryoprobe (QCI). Authors’ spectra just provided for illustration issues.

In tomatoes, incomplete tissue disruption is one of the major sources of variation in a metabolite profiling workflow [31]. For example, tomato skin is notoriously difficult to completely homogenize compared to fruit pericarp tissue [31]. Also, the removal or not of the seeds can interfere with the results since they are more difficult to homogenize. Therefore, it is important to ensure that complete disruption of the tissues has been achieved during extraction [31]. Concerning extraction, it is important to remember that in metabolomics a wide range of different compounds with different chemical natures and solubility ranges has to be analyzed. In tomato, several extraction protocols using methanol-water (for polar/semi-polar metabolites, such as organic acids, carbohydrates, amino acids, quaternary ammonium compounds, and hydroxycinnamic acids), and/or chloroform (for a polar/nonpolar metabolites, such as carotenoids and fatty acids) [34], with variations in the relative amount of each solvent, in one- or multistep processes, and possibly at different temperatures, are described in the literature [35]. In untargeted plant metabolomics, methanol–water mixtures with or without acidification (e.g., formic or acetic acid) have become popular extraction solvents, since they allow extracting a wide range of metabolites [36]. For lycopene extraction, a mixture of methanol and chloroform (80:20, *v*/*v*) was also reported [37]. Moreover, for high-throughput fingerprinting, the recommended procedure is to perform the extraction directly with deuterated solvents, and so, after extraction and centrifugation, the supernatant can be directly transferred into an NMR tube for data acquisition. One drawback is the difficulty to control pH (in fact pD) and ionic force for the samples within a series. Variation in pH between aqueous samples can cause a significant difference in the chemical shifts of signals belonging to metabolites with acidic or basic functional groups (e.g., organic acids and amino acids). A classic case of this issue occurs for the diastereotopic methylene protons in citric acid, which is a major organic acid in tomato, as described by Dona et al. [38]. Changes in pH between samples may alter the ionization of the carboxylate groups in citric acid and thus affect the chemical shift of these methylenic protons and the width of these signals, which may overlap with others in the spectrum. Reproducible chemical shifts can be more easily obtained by using a buffered NMR solution [38]. Nevertheless, citric acid can chelate metal ions such as calcium, magnesium, potassium, and sodium and, even if samples are buffered to a constant pH, changes in metal ion concentrations between samples, may have a significant effect on the chemical shifts and the half bandwidths of the involved signals of citric acid and also any other metabolites with similar properties [38]. Figure 3 shows ^1^H NMR spectra obtained from CD_3_OD: D_2_O KH_2_PO_4_ buffer at pH 6.0 (50:50, *v*/*v*) extracts of tomatoes from Almeria province (Spain) showing these differences in chemical shifts and splitting resolution as a function of the tomato variety. In these cases, special attention must be paid during the process of bucketing of the spectra, and variable-sized binning can be obtained, as it will be later discussed.

#### 2.2.2. Data Acquisition

With respect to NMR acquisition, one-pulse sequence methods are mostly preferred for quantitative NMR profiling of polar metabolites in samples depleted of water. For samples that contain water, the water signal suppression is an important aspect for spectral acquisition and a variety of NMR pulse sequence techniques are available for solvent suppression, such as the ^1^H-nuclear Overhauser effect spectroscopy (noesy), known as noesy-presat. The noesy-presat is the technique of choice for the acquisition of high-quality and reproducible spectra from aqueous samples. It provides a reproducible and easy-to-implement experiment for recording one-dimensional ^1^H spectra of biological samples with good water suppression and has the highest chemical shift selectivity over other presaturation methods [35]. As a result, this pulse sequence has become the predominant approach used by NMR researchers in metabolomics [39]. Other practical considerations includes the addition of an internal standard such as 3-(trimethylsilyl)-2,2′,3,3′-tetradeuteropropionic acid (usually abbreviated to TSP) or deuterated forms of 4,4-dimethyl-4-silapentane-1-sulfonic acid (DSS) or its sodium salt, for aqueous samples, and tetrakis(trimethylsilane)silane (TTMS) for lipophilic samples [38]. After spectra acquisition, automation of phasing and baseline correction can be used, but only with limited reliability, and human visual inspection of processed spectra is always needed to detect artifacts of automated processing.

About data pre-processing, raw analytical data must be processed in a variety of ways to produce comparable lists of metabolites in the samples on which meaningful analysis of treatment differences can be based. Several spectral processing steps are necessary to be applied to raw data in order to permit latter statistical analysis and include: identification and removal of unwanted spectral regions (e.g., residual water signal and solvent signals), normalization (each area of bin is divided by the total spectra area without the excluded spectral regions), binning or bucketing (regular bin size of 0.01, 0.02, and 0.04 ppm, or variable-sized bin) and further scaling (e.g., Pareto, mean centering) [35]. As commented before, even if attention was paid to pH titration, the ionic force of samples in the same batch can slightly differ and variable sized bin can be used to take into account shifts of some signals, e.g., for citric acid [40]. For binning or bucketing of the spectra, commercially available software can be used an include AMIX in any version (Bruker BioSpin GmbH, Rheinstetten, Germany), KnowItAll Metabolomics Editions in any version (Bio-Rad Laboratories Inc., Hercules, CA, USA) or NMRProcFLow package (INRA UMR 1332 BFP, Bordeaux Metabolomics Facility).

#### 2.2.3. Data Analysis

The file containing the intensity values of each bin or bucket could be then transferred to generic statistical software for multivariate data analysis. Common software packages employed in NMR metabolomics studies in plant science are SIMCA-P (Umetrics) or Metaboanalyst (Xia Lab, McGill University, https://www.metaboanalyst.ca/). Multivariate statistical methods such as principal component analysis (PCA) are used to compare sets of spectra to identify clusters of similarity or differences between them, so that conclusions can be drawn about the classification of individual plant samples [41]. Also, supervised methods such as partial least-squares (PLS) and orthogonal projection to latent structures (OPLS) models can find the best fitting relationship between independent and dependent variables. The identities of metabolites responsible for differences between classes can be investigated from loadings plots generated by unsupervised or supervised techniques [21]. The identification of such metabolites is extremely valuable for understanding the underlying biological differences. Combining 1D NMR for quantification and 2D NMR for unambiguous identification of the observed metabolites justifies why NMR is a useful method for plant metabolite profiling, especially of bulk metabolite compounds. Also, metabolite identification is typically achieved by comparison of signals with library data and public and commercial databases, such as Human Metabolome Database (HMDB), Complex Mixture Analysis by NMR (COLMAR) or Chenomx (Edmonton, Canada) [42,43]. Typically, ca. 30 to 100 metabolites are identified, always including some of them that remain unknown analytes after the analysis [25]. Nevertheless, the advantage of a metabolomics study is that assigning a complete 1D ^1^H NMR spectrum is not necessary for a global analysis of the metabolome, but to identify the specific metabolites that are changing and are the main contributors to class distinction.

#### 2.2.4. Data Interpretation

**Figure 4:**
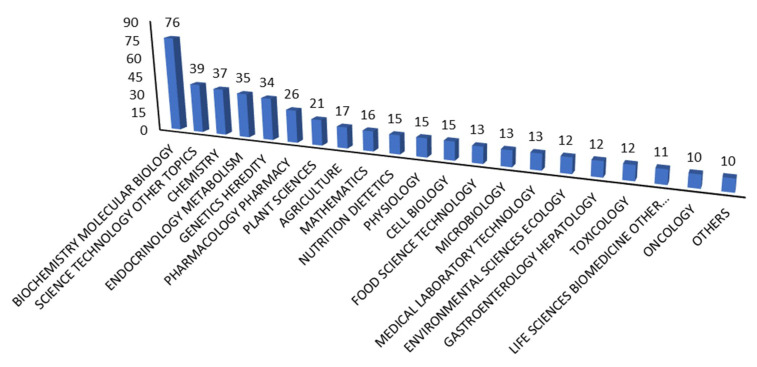
Web of Science search results illustrating the percentage of articles as a function of research area. The searched words were “metabolomics” as topic.

The successful implementation of metabolomics as a discovery or screening platform entails the generation of consistent and reproducible data (identification and quantification) of a broad range of metabolites in a given sample. It culminates with the generation of experimentally testable biological hypotheses, which can serve as the framework for directed and targeted studies and that may open the door to other research questions or to revoke certain paradigms. It is important to keep in mind that metabolomics, while heavily driven by the techniques of chemistry, is fundamentally a study of biology, and the greatest emphasis should be on the biological understanding of data [1]. In fact, out of the 36,313 results obtained on July 2020 in web of science under the search of articles within the topic ‘metabolomics’, an overall of 27,656 (76%) belonged to the research area of biochemistry and molecular biology, whereas only a 37% corresponded to the chemistry field (Figure 4).

Metabolic pathway and cluster analyses of metabolites may be a valuable way to determine relationships between the various metabolites, thus identifying common pathways [44]. Metabolites derived from the same precursors should group together. Hierarchical cluster analyses are often used to observe the variation rules of the significant differential metabolites. The construction, interaction, and pathway analyses of potential biomarkers may be performed with MetaboAnalyst software, with database sources, including the METLIN tandem mass spectrometry (MS/MS) and the Kyoto Encyclopedia of Genes and Genomes (KEGG), to help identifying pathways that were most significantly altered. The KEGG is the main public database that not only provides all possible metabolic pathways but also a comprehensive description of the enzymes that could catalyze each step of a metabolic reaction [45]. Metaboanalyst software allows users to perform Metatolite Set Enrichment Analysis (MSEA), and metabolic pathway analysis (including pathway enrichment analysis and pathway topology analysis) for 21 model organisms, with a total of 1600 pathways, and also to simultaneously analyze genes and metabolites of interest within the context of metabolic pathways [46]. Also, the MetPA (Metabolomic Pathway Analysis) software is a web-based tool that derives much of its power from the KEGG metabolic pathways database.

## 3. Applications of NMR-Based Metabolomics in Tomato

The importance of tomato as a component of the human diet has made it the most studied fruit. As any other fruit, tomato fruit components can be ubiquitous primary metabolites (such as amino acids, sugars, and organic acids) involved in the basic functions of the living cells or secondary metabolites that are usually fruit-type specific. The chemical composition of a tomato fruit is dependent on innumerous factors, some them could be the type of cultivar, climatic and environmental conditions (light, temperature, humidity, atmospheric CO_2_, and air pollutants), agronomic practices (soil properties, water quality, mineral nutrition, salinity, grafting, pruning, growing systems, growth promoters, maturity, and mechanical and pest injuries), stage of ripeness at harvest, and postharvest practices, i.e., appropriate handling and conditioning all the way from the field to the consumer [47,48,49]. The improvement of both organoleptic and nutritional properties of tomato requires identifying, analyzing and interpreting the significance of all these changes on tomato chemical constituents that may undergo during plant growth, development, and by the perturbations imposed on the plants [50].

**Figure 5:**
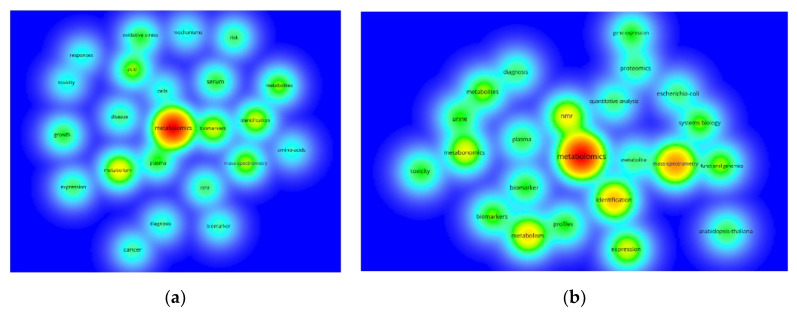
Vosviewer density maps illustrating the co-occurrence of keywords in articles of (**a**) 2020 and (**b**) 2010, containing the word ‘metabolomics’ in article titles.

Applications in the food industry regarding, for example, tracing and tracking and food adulteration, to disclose sophisticated frauds or address the geographical origin, as well as to reveal potential markers for other authentication purposes, have been described. However, perhaps the greatest area of application has been in the general field of plant breeding where different technologies are already being exploited to expand further our knowledge on how the composition of plant products are influenced by genotype and environment. Despite the majority of the metabolomic studies on tomato research using MS techniques [11,51,52,53], NMR is paving its way has a predominant untargeted metabolomic profiling method in this field. As a matter of fact, during the current year, 644 articles contain the word ‘metabolomics’ in their title, and from all those a density map of keywords co-occurrence has been derived with the help of the program Vos viewer (Figure 5). Interestingly, the word ‘mass spectrometry’ appear as keyword in 54 articles, whereas the word NMR shows up only 32 times. If the same exercise is performed within 2010, the snapshot follows a similar trend with 29 times for the former keyword and just 16 for the latter.

In this section, we will review recent advances and applications of NMR-based metabolomics research on tomato as a consolidated untargeted analytical platform that hyphenated with statistical methods increases year by year its application in plant science.

### 3.1. Assessing Fruit Development, Fruit Phenotypes, and Genetic Diversity

Metabolomic strategies have been concerned with obtaining quantitative information on the metabolome of different cultivars of tomato fruit. For example, Moco et al. [54] obtained the metabolic profiles of ripe fruits from 50 different tomato cultivars, including beef, cherry, and round types, by the use of both ^1^H NMR and accurate mass LC-quadrupole time-of-flight (QTOF) MS, to detect a larger range of chemically diverse metabolites, coupled to unsupervised multivariate analysis. Several NMR metabolomic studies also aimed to differentiate the metabolic composition of several tomato cultivars on different tissues and also on different stages of fruit development. In fact, metabolite levels and fluxes have demonstrated large spatial and temporal changes among the different fruit tissues and throughout plant and organ development (e.g., in the accumulation of endogenous metabolites) and even throughout night and day cycles. For instance, it is known that during tomato fruit development there are large changes in the levels of primary metabolites, including carbohydrates and acids, while at the onset of ripening flavonoids and carotenoids begin to accumulate, and the content of the bitter glycoalkaloid, α-tomatine, markedly decreases [4]. Characterization of fruit tissue proportion and composition in a particular tomato cultivar is thus a first step towards understanding the input of the different fruit tissues to its final organoleptic quality. Lemaire-Chamley et al. [55] studied the metabolic regulations between different tissues (seeds, exocarp, mesocarp, columella with placenta, and locular tissue) on tomato fruit, and also during fruit development (12, 20, 35, and 45 days post-anthesis), by the comparison of metabolite networks based on correlations between NMR metabolomic profiles (and other techniques), and revealed compositional similarities with parallel trends along development, but also content differences among tissues (e.g., higher contents of chlorogenate in locular tissue and of starch in columella) that may affect fruit organoleptic quality, including its texture, shape, and taste, especially due to the sugar over acid ratio, and fruit processing quality. Mounet et al. [56] developed a global approach to characterize changes in metabolic profiles in flesh and seeds from the same tomato fruits from 8 to 45 days post anthesis through untargeted (^1^H NMR) and targeted metabolic profiling (LC-DAD or GC hyphenated to flame ionization detector or FID) coupled to chemometric routines. They found that compositional changes were related to physiological processes occurring in each tissue, specifically regarding to: some parallel changes at early stages in relation to cell division and transitory storage of carbon; metabolites participating in the fleshy trait; and metabolites involved in the specific developmental patterns of the seeds [56].

Metabolic changes occurring during tomato ripening are also a subject of interest. Tomatoes experience complex sequential physiological and structural changes during ripening, that include alterations in pigment biosynthesis, decrease in resistance to pathogen infection, modification of cell wall structure, conversion of starch to sugars and increase of the levels of flavor and aromatic volatiles [57]. Sorrequieta et al. [58] studied the changes in the metabolite composition of the tomato fruit (cultivar Micro-Tom) ripened on- and off-the-vine by ^1^H NMR, finding significant differences under both ripening conditions principally in the contents of fructose, glucose, aspartate, and glutamate.

Besides liquid ^1^H NMR, other NMR methods have been used to study tomato ripening. NMR relaxometry and high resolution imaging by NMR or magnetic resonance imaging (MRI) have been applied previously: firstly by Saltveit [59], in 1991, in freshly harvested and of mature-green tomato fruits; by Musse et al. [60], in 2009, on tomato fruit during 3-week periods of postharvest ripening, who detected variations in the transverse (T2) and longitudinal (T1) relaxation times from quantitative MRI images that provided information about all major sub-cellular compartments and water redistribution among them during ripening; by Cheng et al. [61], in 2011, to investigate spatial-temporal changes in sugar and lycopene contents of tomato fruit during ripening in the outer pericarp and columella; by Zhang et al. [62], in 2016, who managed to classify correctly about 86% of the red tomato fruit and 91% non-red fruit when applied a partial least square-discriminant analysis (PLS-DA) model obtained from signal intensities of the pericarp region of acquired MRI images. Additionally, Pérez et al. [50] investigated the feasibility of high-resolution magic angle spinning (HR-MAS) NMR for metabolic studies on the flesh, peel and seeds of tomato (commercial name Zayno from Almería, Spain) at three different ripening stages (green, turning, and red). Chemometric analysis of NMR data revealed a clear separation between seed (increase of triacylglycerols), from peel and flesh (increase of fructose, glucose, citric acid, γ-aminobutyric acid (GABA), glutamine, and glutamate) and allowed the metabolic trajectory from green to red mature stages. ^1^H HR-MAS NMR combines high resolution with a minimal sample manipulation and offers the possibility of analyzing polar and non-polar compounds simultaneously [50], although charging the corresponding NMR rotors and optimizing the acquisition parameters under magic angle spinning are usually time-consuming tasks.

### 3.2. Characterization of Tomato Quality, Origin, and Authenticity

Tomato fruit quality, nutritional value, aroma, taste, and health-promoting effects is a direct function of its metabolite content at harvest. In particular, tomato nutritional quality is highly correlated with the presence of antioxidant and health-promoting metabolites including vitamins C and E, lycopene, β-carotene, lutein, and flavonoids such as quercetin [48]. Variations in early fruit development and composition may have major impact on the taste and the overall quality of ripe tomato [63].

To address tomato quality, it is crucial to know consumers’ expectations and preferences to ensure product success on the market. However, information derived from expert panels, in terms of sensory descriptors, does not provide R&D with enough technical information useful to enhance product features. Such information can only be provided through analytical products evaluation [64]. Therefore, to improve the food chain quality, it is of paramount importance to study the occurrence and concentrations of a small set of metabolites that are predictive for fruit sensorial and nutritional properties and that can be used as potential markers for product quality. Such study can allow for the screening and identification of properties that are traits for breeding (i.e., metabolomics assisted breeding) [65]. Malmendal et al. [64] were able to correlate the NMR metabolomic profiles of canned tomato samples to their sensory descriptors (bitterness, sweetness, sourness, saltiness, tomato and metal taste, redness, and density), suggesting that NMR might be a very useful tool for the characterization of sensory features of tomatoes. Fresh fruit marketability is also linked to shelf-life, which is affected by firmness. Both traits have been shown to be associated with malate content in tomato by López et al. [66], which applied GC–MS and ^1^H NMR to quantify metabolites and a neural network approach to revealed that fruit morphology are strongly associated with specific metabolites such as aspartate, serine, glutamate, and 2-oxoglutarate. The formation of metabolites that are responsible for off-taste formation may also be monitored, so the production and processing practices can be adjusted to prevent the appearance of such metabolites. An example of this is the monitoring of spoilage on tomato sauces which will be discussed later in this Review.

NMR metabolomics approaches also can help to study the occurrence and concentrations of a certain metabolites that can be used as potential markers for product origin and authenticity. For example, Sánchez Pérez et al. [67] found clear differences at a metabolic level, mostly in fructose and organic acids, between “flavor varieties” of high quality tomatoes (the non-hybrid Raf tomato and two hybrid varieties Rambo and Zayno) marketed as Protected Geographical Indication (PGI) from Almería (Spain), by applying ^1^H HR-MAS NMR spectroscopy in combination with PCA and assigned signal analysis (ASA), and also as a function of the ripening process, for which γ-aminobutyric acid (GABA) was envisioned as a good marker for its monitoring. Moreover, they revealed the existence of variety-dependent relationships between external appearance and metabolic content [67]. Similarly, Mallamace et al. [68] successfully applied HRMAS NMR-based metabolomics approach to determine the metabolic profile of a famous Sicilian cherry tomato from Pachino also with a PGI certification of quality, and to predict the origin of a tomato sample, thus avoiding commercial frauds which have been originated in the Italian and international markets.

### 3.3. Compositional and Quality Changes According to Agronomic Practices

Producers need diagnostic tools to follow the organoleptic and nutritional quality of their food products during the season or after modification of their cultivation practices. Deborde et al. [69] studied the effect of two different fertilization practices (with or without recycling of the nutrient solution) in 32 major compounds in two tomato varieties (cv. Palmiro and Clotilde) at harvest during an all season using untargeted ^1^H NMR quantitative profiling. While some seasonal changes in solar radiation were found to affect the sugar/acid ratio (causing a possibly lower organoleptic quality), only citric acid showed a few compositional differences between the two varieties when applying nutrient recycling, which allowed to confirm that nutrient solution recycling is a way to limit the waste of liquid fertilizers. Iglesias et al. [70] applied ^1^H HR-MAS NMR spectroscopy combined to chemometrics tools to compare the metabolic profile of four marmande type tomato varieties from Almería (commercial names Raf, Delizia, Tigre and Rafaelito), when growing under different conditions (traditional sand-covered soil and hydroponic—perlite, New Growing System NGS^®^, and grafting). The authors found some variety-dependent composition changes mainly in the sugars/acid ratios that were produced by climatic conditions, and highlighted that fruits grown in the hydroponic system NGS^®^ showed improved tomato quality, due to a depletion in amino acids and increased content in sugars and citric acid. In addition, they considered malic acid as a good marker candidate to differentiate “Tigre” tomatoes (which showed to be less affected by the date of sampling or culture) [70]. Later, the same group [71] investigated the effect of salinity and silicon treatments on the marketable quality of the same four Marmande tomato varieties through conventional quality attributes by ^1^H HR-MAS NMR spectroscopy to deduce the content of GABA, which was associated to ripening in three varieties. NMR data also lead to a new taste index, which was crucial for understanding the effects of environmental and nutritional factors on the quality of the fruits [71]. Hohmann et al. [72] applied a ^1^H NMR profiling approach for the authentication of organically produced tomatoes analyzing a total of 361 tomato samples of two different cultivars and four different producers during a 7-month period. Although linear discriminant analysis demonstrated good discrimination between organically and conventionally produced tomatoes, unexpected differences between individual producers made the prediction of the cultivation method difficult [72]. Later, the same group optimized the ^1^H NMR classification model by including additional data of isotope ratio mass spectrometry (IRMS) and mid-infrared (MIR) spectroscopy, in which fused data yielded better validation results than individual methods, and allow for the authentication of organically produced tomatoes [73].

**Figure 6:**
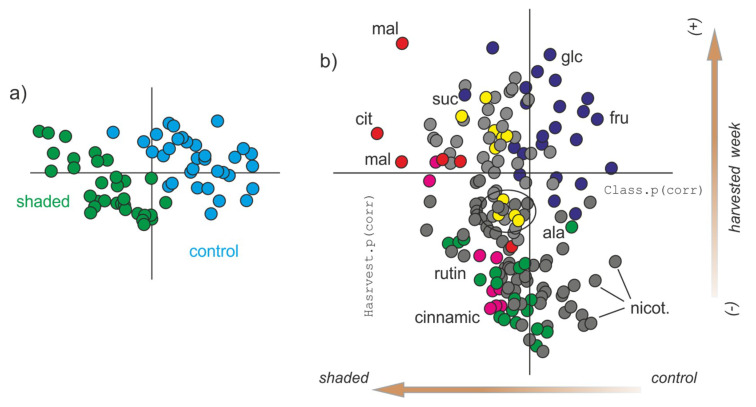
Partial least square-discriminant analysis (PLS-DA) (**a**) scores plot and (**b**) Shared and Unique Structures (SUS) plot adapted from Abreu et al. [37]. The SUS-plot clusters the loadings according to their importance [*p(corr*)] for the discrimination of the NMR data with the harvest week (Y axis) and with the light condition (X axis, control, and shaded conditions on the right and left sides, respectively), showing that fructose and glucose increased in control tomatoes, while sucrose, cinnamic acids, rutin, fatty acids, most amino acids, and organic acids (malate and citrate) increased in shaded tomatoes.

Some authors have also applied NMR metabolomics to study the effect of climatic parameters, such as solar radiation and temperature, on tomato chemical profile. Masetti et al. [74] applied ^1^H NMR spectroscopy coupled to chemometric tools to assess variations in the lipophilic content of two varieties of PGI Pachino cherry tomatoes caused by climatic factors, e.g., solar radiation and average temperature, over a period of 3 years. They showed that the metabolic profile was differently affected by climatic conditions for the two varieties and that the compounds mostly affected by cropping period were *α*-tocopherol, unsaturated lipids, chlorophylls, and phospholipids. Bénard et al. [75] studied the diurnal compositional changes in tomato (*Solanum lycopersicum* cv. Moneymaker) leaves and fruits of plants cultivated in a commercial greenhouse experiencing different light regimes (cloudy vs. sunny day, and control vs. shading condition) by applying a high-throughput robotized biochemical phenotyping of major compounds, as well as ^1^H NMR and MS metabolomic profiling, together with multivariate, univariate and clustering analyses and correlation networks. Their results indicated that the shaded carbon-limited plants decreased their sink carbon demand and showed subtle compositional modifications along a diel cycle, including those directly linked to photosynthesis and photorespiration (in leaves) and, in a lesser extension, in several organic acids (in fruits) [75]. Similarly, Abreu et al. [37] analyzed the metabolic profile of Almería greenhouse organic tomatoes (*Solanum lycopersicum* cv. Delyca) fruits and leaves under different light regimes produced by a shade mesh (control vs. 50% reduction of sunlight radiation). Metabolic profiling achieved by NMR spectroscopy in the fruits allowed to conclude that a shading mesh resulted in a reduction in tomato production and in smaller fruits with lower contents of sugars and carotenoids and higher contents of organic acids, amino acids, polyunsaturated fatty acids (linoleic and oleic acids), phenylpropanoids, and flavonoids (which contributed to an increased antioxidant activity) (Figure 6).

**Scheme 1:**
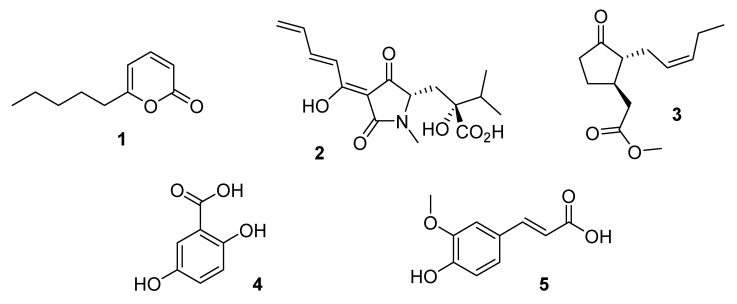
Molecular structure of 6-pentyl-2H-pyran-2-one (**1**), harzianic acid (**2**), methyl jasmonate (**3**), gentisic (**4**), and ferulic acid (**5**).

Studies addressing plant metabolic changes triggered by certain agronomic practices have been also described. For example, by applying HR-MAS NMR spectroscopy, Mazzei et al. [76] found that tomato leaves of plants supplemented with two secondary metabolites released by Trichoderma fungi, 6-pentyl-2H-pyran-2-one (1) and harzianic acid (2), to improve plant growth and health, showed significantly enhanced acetylcholine and GABA content accompanied by variable amount of amino acids (Scheme 1).

### 3.4. Characterization and Detection of Unintended Effects in Genetically Modified Crops

Tomato breeding began in Europe when improved cultivars were generated to meet several needs including fresh market and processing industries [77]. Since then, vast research and economic efforts have been invested in tomato productivity improvement and to boost food production in the world. These practices, however, have resulted in the loss of some 95% of the genetic and chemical diversity of wild relatives, and also in a decrease in flavor of tomato fruits over the last decades, according to consumers. Modern breeding specifically focuses on developing varieties that incorporate multiple disease-resistant loci that are introgressed from wild relatives, show enhanced crop productivity, and that rescue desirable nutritional and organoleptic traits [78].

Regulatory bodies are placing much emphasis on the identification of unintended effects of genetic modification (GM) and there has recently been a drive to establish methods of analysis to screen them. NMR fingerprinting combined with chemometrics and univariate statistics can successfully trace even small differences in metabolite levels between plants and therefore represents a powerful tool to detect potential unintended effects in genetically modified crops. Perez-Fons et al. [79] described a multi-platform metabolomic analysis, using NMR, MS, and HPLC-UV/Vis to describe introgression lines of a wild tomato species (*Solanum pennellii*) with a domesticated line in order to analyze and quantify alleles (QTL) responsible for metabolic traits. They have identified QTL for health-related antioxidant carotenoids and tocopherols, as well as molecular signatures for some 2000 compounds. By applying off-line ^1^H LC-NMR, Noteborn et al. [80] only found minimal compositional differences between tomatoes from isogenic transgenic (variety transgenic antisense-exogalactanase Moneymaker tomato) and wild-type lines and these were postulated to be false positives due to changes that arose from natural variability and/or external factors such as intra-fruit variations, stages of ripening, etc. Le Gall et al. [81] could overexpressed specific maize transcription factors in tomato (*LC* and *C1*) to increase their flavonol content, and statistically significant changes in 15 metabolites were detected when compared with non-modified controls grown side-by-side under the same conditions. Also, PLS of NMR data showed clear separation of samples according to ripening stage and genotype, due to changes in the levels of glutamic acid, fructose, and some nucleosides and nucleotides, which gradually increase from the immature to the ripe stage, whereas valine and GABA were present in higher amounts in unripe tomatoes [81]. Moing et al. [82] applied ^1^H NMR to obtain the metabolic profiling of soluble sugars, organic acids, amino acids, and some secondary metabolites in fruit, roots, and leaves of wild types and tomato transformants overexpressing hexokinase. PCA of metabolic profiles revealed that environmental factors, i.e., culture conditions, can significantly modify the metabolic status of plants and thus hide or emphasize the expression of a given genetic background.

### 3.5. Study Post-Harvest Processes in Tomato Fruits

Post-harvest losses of fruit and vegetables are significant and therefore stemming such losses has been an on-going activity among horticulturalists and geneticists. Ethylene regulates a myriad physiological and biochemical processes in ripening fruits and is accepted as the ripening hormone for the climacteric fruits. However, its effects on metabolome and resulting fruit quality are not yet fully understood, particularly when some of the ripening-associated biochemical changes are independent of ethylene action.

Studies applying NMR metabolomic approaches to study post-harvest processes in fruits and vegetables are few and have been carried out mostly on tomato. Some of these have been mainly devoted to the exploration of the metabolomics pathways of the fruits through the silencing of genes that impede the production of ethylene. The analysis of profiles of metabolites by NMR has been carried out on transgenic tomatoes designed to accumulate polyamines (spermidine and spermine) [83,84,85], described as potent inhibitors of senescence and maturation processes in different plant tissues, and on tomatoes deficient in the production of ethylene [85]. With respect to tomatoes that accumulated polyamines, several studies agree that they also up-regulate the accumulation of metabolites such as glutamine, glutamate, asparagine, aspartate, GABA, citrate, and fumarate [83,84,85]. Moreover, transgenic tomato fruits showed higher fructose/glucose and sugar/acid ratios, as revealed by Mattoo et al. [83], suggesting that the pathways involved in the nitrogen sensing/signaling and carbon metabolism seem preferentially activated in these transgenic fruits, and an increase in lycopene accumulation and in ethylene production, as observed by Neily et al. [84], suggests that a high level of accumulation of polyamines in the tomato regulates the steady-state level of transcription of genes responsible for the lycopene metabolic pathway. Kausch et al. [86] developed transgenic tomato fruits that, contrarily to the previous authors, had severely reduced methyl jasmonate levels 3 (Scheme 1) (that lowered polyamine levels) and, in accordance, found a decrease in the accumulation of all the above-mentioned metabolites that were observed to increase in higher-polyamine tomatoes, among other metabolites changes. Sobolev et al. [85] have generated a homozygous transgenic tomato genotype (2AS-AS) that exhibits reduced ethylene production, which had a longer shelf life, and crossed it with high polyamine tomato line thus generating a double transgenic hybrid (2AS-AS × 579HO) that resulted in significantly lower ethylene production. Ethylene deficiency resulted in decreasing levels of most amino acids except aspartate and glutamate compared to the control line and had higher phenylalanine levels compared to high polyamine fruits.

Neelam et al. [87] studied the metabolic composition of field grown wild and transgenic tomatoes engineered to accumulate spermidine and spermine but in three alternative farming practices: standard black polyethylene bed, and two cover crop based sustainable systems using leguminous hairy vetch (*Vicia villosa*; HV) or non-leguminous rye (*Secale cereale*). A surprising synergy was found between transgene expression and HV cover crop cultivation system, since HV activates the same metabolic pathways and genes as those activated by the introduction of the ySAMdc transgene. Besides HV also modulates transgene expression and actually affects the pattern and quantity of metabolites during fruit ripening [87]. Fatima et al. [88] also demonstrated distinct effects on tomato metabolome caused by the abundance and deficiency of plant growth regulators, such as ethylene, methyl jasmonate (**3**) and polyamines generated via transgenic intervention, and by the agroecosystem environment (hairy vetch, rye, plastic black mulch, and bare soil mulching systems). They applied NMR spectroscopy coupled to univariate/multivariate methods and created networks with covariances of responses that depicted changes in correlations of paired metabolites. Important differences were found in the primary fruit metabolome of tomato transgenic lines grown in a temperature and light controlled greenhouse. For example, under field conditions high polyamine lines were richer in amino acids and energy metabolites than was under greenhouse conditions, while organic acid levels did not differ. Also methyl jasmonate-deficient fruits grown in the greenhouse were remarkably different with higher concentrations of polyamines and lower levels of many amino acids [88].

### 3.6. Impact of Abiotic and Biotic Stress

Early economic losses occur in the agricultural industry worldwide due to insects and pests. The monitoring of health and detection of various infections in plants is therefore critical for sustainable agriculture. Detecting early information on crop health and pest infestations can facilitate the control of these often devastating infections through proper management strategies such as vector control with the application of pesticides, fungicides and specific chemicals that can improve productivity [89]. Metabolomics allows for the identification of biochemical markers that have important roles in plant resistance to pests and diseases. This knowledge can help breeders to select plants based on differences in such compounds. The studies reported in this section are more focused on tomato plant metabolic alterations and not so much in the fruits.

The metabolite modifications in tomato roots and leaves in response to cadmium (Cd) stress was investigated through ^1^H NMR-based metabolomics approach by Zoghlami et al. [90], in plants exposed for 3 and 10 days to 0–300 μM CdCl_2_ concentrations, and by Hédiji et al. [91], in plants exposed for 90 days to 0–100 μM CdCl_2_. Zoghlami et al. [90] found that several amino acids significantly accumulated after 10 days of Cd exposure, mostly asparagine, which increased by 26 fold in the roots of 300 μM Cd treated plants, being considered a good marker for Cd stress, and tyrosine which content was increased by 10 fold in leaves after three days of treatment with 30 μM. The results were supported by Hédiji et al. [91] work, in which Cd-treated leaves showed an increased in asparagine and tyrosine and also in α-tocopherol accumulation and a reduction in proline and total ascorbate.

With respect to plant-mediated responses to biotic stresses, ^1^H NMR spectroscopy combined with chemometrics tools have been applied by several authors to analyze the metabolites involved in the tomato plant defense against nematodes [92], tomato mosaic virus (ToMV) [93], thrips [94], leafminer (*Tuta absoluta*) [95], and *Citrus exocortis* viroid (CEVd) [96]. Afifah et al. [92] reported that glucose and caffeic acid, which is one of the phenolic compounds claimed to have a negative effect on nematodes, significantly increased in resistant tomatoes. López-Gresa et al. [93] observed metabolic alterations due to plant development (general decrease in organic acids, amino acids except asparagine, phenylpropanoids, and rutin), position of the leaf (higher levels of organic acids, some amino acids, phenylpropanoids, and flavonoids in lower leaves while elevated amounts of sugars were present in the upper ones), plant defense response to virus inoculation (while flavonoids accumulated in virus-inoculated leaves, increased levels of phenylpropanoids were observed in non-inoculated leaves where ToMV actively replicates), and harvesting time (an increase of amino acids and organic acids, except glutamic acid, were observed in samples collected in the morning, whereas sugars and secondary metabolite levels increased in the tomato leaves harvested in the evening). Mirnezhad et al. [94] found that only wild tomatoes were thrips-resistant, and that thrips-resistant tomatoes contained acylsugars, which are known for their negative effect on herbivores. de Falco et al. [95] found that GABA was relatively much higher in all infested samples of three different tomato genotypes compared to the non-infested plants, and that infested genotype samples were rich in organic acids, including fatty acids and acyl sugars, chlorogenic acid, neo-chlorogenic acid and feruloyl quinic acid [95]. Lopez-Gresa et al. [96] observed that glycosylated phenolic compounds, including gentisic (**4**) and ferulic acid (**5**) (Scheme 1), accumulated in CEVd-infected tomato plants, together with some amino acids (phenylalanine, tyrosine, aspartate, glutamate, and asparagine). Also, the authors observed that GABA was the only metabolite that significantly accumulated in benzothiadiazole-treated tomato leaves, thus they have tested GABA addition on tomato plants infected by CEVd, and a reversion of the NahG hyper-susceptibility to CEVd was observed, indicating that GABA could regulate the resistance to CEVd induced by benzothiadiazole (BTH) [96].

### 3.7. Metabolomics in Tomato-Derived Products

So far, we have seen how recently NMR is largely used as valid and prominent tool in metabolomic applications in tomato fruits and plants. Studies on the application of NMR-metabolomics approaches to evaluate tomato-derived products can also be found. For example, Consonni et al. [97] investigated the metabolite content of a total of 119 Italian and Chinese tomato paste of two production years (2007 and 2008) by ^1^H NMR and multivariate data analysis tools, with the aim of building a robust geographical differentiation statistical model. Bidirectional orthogonal projection to latent structures-discriminant analysis (O2PLS-DA) was capable of predicting the geographical origin of all analyzed samples, regardless of the year. Sobolev et al. [98] characterized and compared tomato juices and pulps from two tomato cultivars by liquid and magic angle spinning NMR, proving that all the water-soluble substances present in the pulp are extracted in the juice and that both liquid-state and HR-MAS NMR produce consistent results concerning the water-soluble fraction, but HR-MAS is able to detect also some insoluble compounds such as lipids. Interestingly, they applied DOSY to a sample of Red Setter tomato juice allowing the differentiation of more than one molecular entity within the same chemical shift.

Also, NMR spectroscopy, as a non-invasive approach, is receiving recent attention in studying problems in industrial process environments, more specifically, to track factory relevant tomato paste spoilage. Pinter et al. [99] found that spoilage in tomato paste test samples leads to longer longitudinal relaxation times T_1_ over a five days room temperature exposure period using a conventional low magnetic field NMR system and prompted the work to be extended to the study of industry standard, 1000 L, non-ferrous, metal-lined totes. The results of this work suggest that a handheld NMR device (e.g., NMR Mobile Universal Surface Explorer, developed at Rheinisch-Westfälische Technische Hochschule (RWTH)-Aachen) can be used to study tomato paste spoilage in factory process environments [99].

The emergence of the field of nutri-metabolomics, which englobes metabolomics platforms to discern person-to-person variations in health and nutritional responses, can also be found with tomato-related products. For example, differences in the origin and ripening stage of fruits might have a strong impact on their phytochemical composition, and consequently, on their potential nutri-metabolomics effects on health. The effects of a 4-week cross-over nutritional intervention with two tomato sauces differing in their natural lycopene content was evaluated on the metabolic status of the serum of 24 young healthy subjects by ^1^H NMR, Bondia-Pons et al. [100]. The study evidenced that the levels of creatine, creatinine, leucine, choline, methionine, and acetate in aqueous extracts were increased after the intervention with the high-lycopene content sauce, while those of ascorbic acid, lactate, pyruvate, isoleucine, and alanine were increased after the normal-lycopene content sauce, and eventually points out that the reason might partly due to the different ripening state of the fruits used in production of the employed tomato sauces.

## 4. Final Remarks

Tomato is an essential crop in terms of economic importance and nutritional quality. Studying tomato metabolic changes may be a rough task, due to the large number of constituents with different physicochemical properties and stabilities and the wide number of factors that affects tomato composition, such as variety, gene modifications, environmental and cultivating effects, conditions, ripening stage and post-harvest storage. Also, NMR knowledge concerning the metabolic shifts underlying these processes remains largely confined to primary metabolism and information is lacking regarding secondary or specialized metabolites [101].

In this review, we have briefly described the impact of NMR-based metabolomics up to the present and its potential to study innumerous aspects related to tomato quality and production, especially in fields as agronomy, stress-resistant varieties, improvement of plant fitness, improving crop yields, plant breeding, among others. Plant metabolomics is a field of science which is still in a dynamic phase of development. Advances in analytical instrumentation and statistical methodologies to make NMR metabolomics more resolutive, more sensitive, and more accessible, are constantly made and could act as real game changers for metabolomics in the near future and contribute to an increasing role of NMR in the “omic” world at the beginning of the 2020s. Also, combination of metabolomics information with transcriptomics and proteomics is crucial and may have the potential to generate a much more complete picture of the composition of food products, to optimize crop trait development, to enhance diet and health, and to help advance plant breeding strategies and the speed of developing new varieties more suited to current demands, especially those related to consumer preferences for taste and nutritional value.
